# Full spectrum town halls for hidradenitis suppurativa: A model for advancing clinician–patient–researcher engagement in clinical and translational research

**DOI:** 10.1017/cts.2025.62

**Published:** 2025-04-11

**Authors:** Anuradha Hashemi-Arend, Michelle A. Lowes, John W. Frew, James Krueger, Athena Gierbolini, Monisa Nayim, Melissa Samanoglu, PaMalick Mbye, Fahim Shahriar, Jonathan N. Tobin, Rhonda G. Kost

**Affiliations:** 1 The Rockefeller University, Center for Clinical Translational Science, New York, NY, USA; 2 The Hidradenitis Suppurativa (HS) Foundation, Cary, NC, USA; 3 University of New South Wales, Liverpool Hospital, Liverpool, NSW, Australia; 4 Clinical Directors Network (CDN), New York, NY, USA

**Keywords:** Patient experience, lived experience, reducing stigma, empathy for rare disease, patient advocacy

## Abstract

Hidradenitis suppurativa (HS) is a chronic, painful inflammatory skin disease affecting 0.1% of the US population. Limited understanding of HS biology and ineffective treatments leave patients dissatisfied, facing misdiagnosis, and diagnostic delays. To address these challenges, the Rockefeller University Center for Clinical Translational Science, Clinical Directors Network, and the HS Foundation launched an initiative to foster engagement among stakeholders. Three full spectrum town halls (FSTH) were designed to engage patients, scientists, and clinicians bidirectionally. Topics spanned the translational research spectrum to amplify patient testimony, update the HS community on scientific and clinical research advances, and promote patient-centered research and care. The FSTH model aims to enhance empathy, foster trust, accelerate scientific discovery, and improve care. FSTH-2018 showcased patients’ experiences living with HS, the scientific and clinical state of the art, and tailored a new HS study to patient preferences. FSTH-2021 shared results of the study, including new insights into HS biology. FSTH-2023 highlighted best practices for outpatient surgical care of HS. Participant feedback underscored FSTH’s role in nurturing empathy and advancing discovery and patient engagement. FSTH serves as an effective model for uniting stakeholders, bridging gaps in knowledge and trust, and accelerating translational research to improve HS care.

## Introduction

Hidradenitis suppurativa (HS) is a painful, chronic inflammatory skin disease [1]. Using US medical claims data, the prevalence of HS has been estimated to be 0.1% [2]. When this project began, the understanding of the genetics [3] and biology [1] of HS was limited, and existing treatment options were not effective for many individuals living with HS [1,4]. More recently, there has been progress in research and treatment of HS using biologics [5].

In a multinational survey of ∼1300 individuals diagnosed with HS, 61% rated HS-related pain in the prior week as moderate or high, and only 9% described having no pain over the past week [6]. Participants described experiencing symptoms of drainage (72%), odor (54%), and fatigue (61%) in the past week and experiencing a flare daily (23%), weekly (30%), or monthly (31%) [6]. Most participants (64%) visited a medical provider more than five times before receiving an HS diagnosis; it took participants an average of 10 years from the onset of symptoms to diagnosis [6]. Almost half (46%) reported feeling dissatisfied with the current treatment [6]. Existing research recognizes a relationship between provider empathy and patient trust and its impact on healthcare [7–9].

The lack of knowledge about HS among medical providers leads to misdiagnosis and diagnostic delays and, combined with a lack of empathy and respect, contributes to mistrust of the medical establishment among HS patients. These factors discourage patients from seeking healthcare and participating in research [10].

HS disproportionately affects underserved populations [10]. A retrospective analysis of more than 48 million patients across the USA used electronic health record data to identify 47,690 patients with HS [2]. While overall HS prevalence was 98 per 100,000 persons, the rate was triple for African Americans (296 per 100,000) [2]. A retrospective study of two cohorts of HS patients found higher disease severity among Black and Hispanic patient populations compared to White patients [11]. In addition, nonWhite patients face greater diagnostic and treatment delays [12] and are underrepresented in clinical trials [13]. A 2023 review of clinical trials on HS found that Black, Hispanic/Latino, and American Indian or Alaska Native patients constituted only 13.7%, 7.2%, and 1.3% of the trial population, respectively [14].

The gaps in understanding of basic HS biology, paucity of treatment options, and alienation felt by HS patients are critical barriers to effective clinical translational research to address this painful, debilitating disease. We sought to encourage patient engagement with scientists and clinicians to bridge barriers, foster trust with the HS community, and accelerate work across the translational research spectrum.

The HS Foundation, founded in 2005 to improve the lives of people affected by HS, conducts education, research, and advocacy through programs for patients, medical professionals, and researchers. They provide online courses and resources, fund research, and advocate for patients to improve HS treatment.

Since 2009, The Rockefeller University Center for Clinical and Translational Science (RU-CCTS) has worked with Clinical Directors Network (CDN) [15], a primary care practice-based research network (PBRN) designated by the Agency for Healthcare Research and Quality (AHRQ) as a Center of Excellence (P30) in Primary Care Practice-Based Research and Learning, to align basic science investigation with community-engaged research priorities [15,16]. The Rockefeller-CDN Community-Engaged Research Navigation (CEnR-Nav) program brings together a range of stakeholders (including basic scientists, community clinicians, patients, and their advocates) to develop interdisciplinary research teams that engage in bidirectional communication. These teams form sustainable partnerships to address scientific and community aims across the translational research spectrum by joining the infrastructure of a Community Engaged Research Core at a Clinical Translational Science Award (CTSA) site with a PBRN [17–20].

This background and expertise uniquely situated RU-CCTS and CDN to partner with the HS Foundation to undertake a series of virtual town hall meetings, full spectrum town halls (FSTH), explicitly designed to address the translational gaps in HS research and treatment by bringing together partners from across the translational spectrum. This report describes three HS FSTHs conducted jointly with HS patients, scientists, and other stakeholders as a model of community engagement that could be replicated for other health topics and offers a useful strategy for institutions to engage with patients and practitioners to accelerate discovery.

## The full spectrum town halls

**Figure 1.**
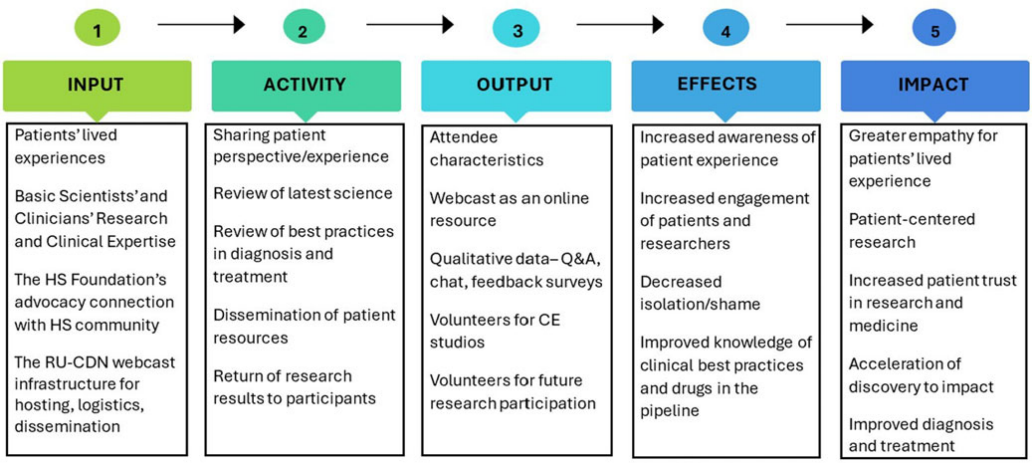
The causal pathway for hidradenitis suppurativa (HS) full spectrum town halls is a logic model that maps how the expertise of HS patients, clinicians, and scientists (resources) contributes to the activities both during and resulting from the town hall, leading to outputs such as publicly archived webcasts and qualitative feedback. The pathway next maps how those outputs lead to increased interdisciplinary understanding among patients, scientists, and clinicians that ultimately impacts participants’ experiences with healthcare, drives more patient-centered research design, and accelerates discovery. Ideally, the outputs, effects, and impacts of a town hall will be disseminated and incorporated into the design of successive town halls. RU = Rockefeller University; CDN = Clinical Directors Network; Q&A = questions and answers; CE = community engagement.

From 2018 to 2023, RU-CCTS, CDN, and the HS Foundation jointly hosted three 60-minute virtual FSTH that addressed the state of the field and advances in mechanistic and clinical research and provided a forum for HS patients to share their lived experiences and engage with translational researchers. In contrast to conferences aimed at scientists or clinicians or exclusively for patients, FSTH are designed to bring together different interested and affected groups – scientists, clinicians, patients, caregivers, advocates, pharmaceutical industry partners, policymakers, and others – to share updates from multiple perspectives in accessible language, foster broader understanding, and accelerate research. The causal pathway (Fig. 1) maps out the logic model for this series of FSTH.

Overall approach: For each FSTH, collaborators from RU-CCTS, CDN, the HS Foundation including the patient advocate, and other key stakeholders (e.g., surgical colleagues) formed a committee to develop the focus and agenda for the meeting and recruited a panel of patients to participate in the agenda. Organizers used three approaches to recruit HS patients to participate in the town hall patient panels: (1) HS patients who were engaged with the HS Foundation were invited to share their stories; (2) physicians in the organizer’s local HS treatment network identified HS patients who might be willing to share their experience of living with HS; and (3) at the time of registration, several HS patients spontaneously offered to share their lived experience of HS. The patient volunteers were then interviewed briefly, the town hall explained, and if they were comfortable to proceed, they were invited to participate. The RU-CCTS community engagement staff reviewed the draft slides for scientific and clinical presentations to ensure the language would be accessible to the public.

The audience for each FSTH was recruited using multiple approaches. The HS Foundation led outreach to their HS patient community and network of patient advocates through their email listserv and by word of mouth. RU advertised the events via email and flyers, inviting scientists, physicians, researchers, medical providers, and hospital administrators. RU community engagement staff shared event notices with HS advocacy leaders who promoted the event to their members’ platforms on social media. CDN sent notifications to their membership of over 50,000 online learners, including clinicians and staff in Federally Qualified Health Centers and other primary care practices, as well as primary care PBRNs, health departments, and the community-engaged research cores of Clinical and Translational Science Awardees CTSA. FSTH speakers and organizers shared announcements through their own online and professional networks and listservs.

## Program and activities of HS full spectrum town halls (FSTH) 2018–2023

**Figure 2.**
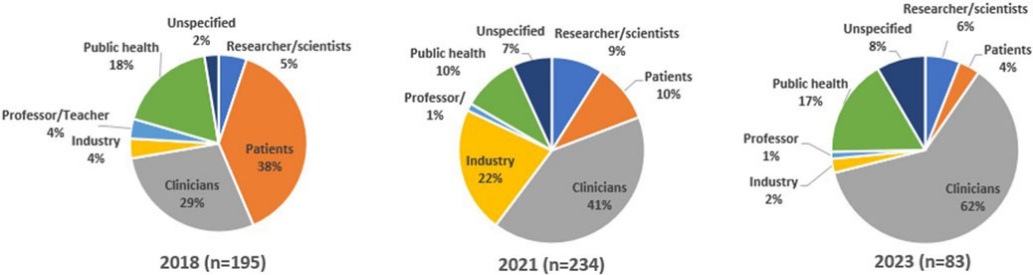
Hidradenitis suppurativa (HS) full spectrum town hall registrants in 2018, 2021, and 2023 by occupation. During online registration, individuals provided information about their occupations, areas of interest, interest in future research participation, and willingness to be contacted. Limited information was elicited about who might also be an HS patient.

I. The first FSTH on HS was held in **2018**, titled ***“Shedding Light on Hidradenitis Suppurativa (HS”)***, with the intention of building a relationship with the HS patient community, whom RU investigators had not worked with previously. One hundred and ninety-five people registered for the FSTH, of whom 38% were patients. Other attendees included medical providers, researchers, public health workers, and teachers (Fig. 2). The first event was hybrid in person and online. Forty-five people attended in person, and 143 attended online. Participants viewed the webcast from 39 states and 6 countries.

Scientific and clinical experts on HS presented what is known about the causes, symptoms, and existing treatments for HS, speaking directly to the audience using nontechnical language and visuals. Presentations included encouraging messages to counter the shame, stigma, and misinformation experienced by many patients. Patients were encouraged to advocate for their needs.

Physician-scientists described what it means to use a translational science approach to investigate skin disease as a partnership between patients with a disease and the researchers who study the disease in both the laboratory and practice-based setting. The approach uses an iterative process between “bedside to bench” and “bench to bedside” science. In “bedside to bench” science, samples are collected from patients presenting a disease in a practice-based setting or hospital (biopsy, skin and tissue samples, etc.) and are studied in the lab to understand the mechanisms of the disease. These insights can then be brought “bench to bedside” through the development and testing of novel or repurposed therapeutics. This mirrors the successful approach previously used by one of the scientists (JK) to unravel the biology and treatment of other skin diseases [21,22]. The team announced a new initiative by the RU Laboratory of Investigative Dermatology to target and define the biologic and immunologic mechanisms driving HS and to conduct translational studies to advance progress toward effective treatments. Investigators highlighted the critical role of patients and invited them to partner in research initiatives.

HS advocate and speakers in a patient panel related their personal experiences living with HS, the trauma of suffering from decades of painful symptoms, ineffective treatments, and misdiagnosis, the relief of an eventual correct diagnosis, and the continued frustration with treatment efforts that have varying levels of efficacy and success. Advocates from the HS community shared resources for raising awareness, seeking support, and community and mental health resources for patients suffering from HS to combat the psychosocial impact of the disease. Patient attendees with HS were very active in the webchat, spontaneously sharing frustrations and experiences in parallel with the speakers (Table 1). They related their experiences and connected over shared symptoms, personal experiences with various treatment options, being misunderstood by medical providers, the impact of HS on personal relationships, and the shame and stigma of HS. Program staff reviewed webchat comments for common themes (Table 1). Additional participant comments are provided in Supplementary Table S1.

**Table 1.** A qualitative review of participants’ comments from the 2018 full spectrum town hall (FSTH) and recurrent themes

Participant comment	Comment-themes	Themes
“I got told it was hygiene so many times! I felt so guilty about feeling like I did this to myself”	Overemphasis on individual behavior (blame)	**Gaps in physician knowledge**
“I definitely had to teach my [primary care] doctor about HS”	Lack of awareness about HS among medical providers	
“YES! The PATIENTS know! Listen to them” “Doctors need to hear this”	Need for empathy for the patient experience	
“20 years before diagnosis for me (and I never talked about it. I was so ashamed)” “I wasn’t even Dx’d until last year – given antibiotics since 1987”	Misdiagnosis and diagnostic delays	
“It took 9 years before I told my family. I was so ashamed and embarrassed”	Shame	**Stigma and the psychosocial impact of HS**
“I stopped having sex 3 years ago when my HS went ballistic. . . shame & embarrassment”	Barrier to intimate relationships	
“I’m so tired of hating my body and feeling like a gross rotting mass”	Frustration with recurring symptoms	
“I had some surgery; never again…worked in the area then it just traveled to other body parts. Not fun.”	Frustration with treatment side effects	
“I had surgery on my arm. Long recovery time (about 8 months) because my skin graft failed”	Cost and experience with different treatment options	**Treatment and symptom management**
“Focused on quitting inflammatory foods now, I’ve quit a LOT of foods, almost ready for AIP (autoimmune protocol)”	Patient reported outcomes of alternative therapies	
“How does diet play a role? I went vegan and it’s working wonders”	Interest in dietary and lifestyle interventions	
“Watching and listening to you [patient advocate] today, will make it easier for me to talk about my own HS with my family and my doctors”	Empowered to advocate for own health	**Impact of town hall speakers**
“Thanks to the fantastic panel of speakers. So exciting to see the medical community coming together and trying to solve the HS problem… we have come a long way in the last few years”	Hope in direction of HS research/treatment	
”I’m so glad I participated in this and thank you ALL for sharing your stories – this is confirmation to me that – I AM NOT ALONE. WE ARE NOT ALONE.” “[patient advocate] is so right” “yes [patient advocate], been there and done that for so long” “Thank you especially for recognizing pain as a primary (and undertreated) complaint!” “I can relate to the speaker. She’s speaking to my heart.”	Empathy/validation of patient experience	

HS = hidradenitis suppurativa; Dx = diagnose.

A final Q&A session opened discussion among patients, clinicians, scientists, and members of pharmaceutical companies.

After the 2018 FSTH, 49 attendees completed an evaluation (26%). Of those respondents, 91% answered said they *Agreed or Strongly Agreed* that “the event was informative,” 83% *Agreed or Strongly Agreed* that the event met their expectations, and 87% *Agreed or Strongly Agreed* that they were “likely to attend another event like this one.”

The 2018 FSTH laid the groundwork for a Community Engagement Studio (CES) that followed to engage patients in refining an HS translational research protocol under development [23]. FSTH audience members who volunteered to work with HS researchers were invited to participate in a CES. Ten individuals – men, women, one adolescent, and her parent – attended the studio. Participants were excited about research participation as a way to make progress on their disease. They expressed concern that patients might feel self-conscious about the exposure of their HS scars and lesions when undressed and asked for proactive draping, which the PI then incorporated into the study procedures. The adolescent female patient said she would feel more comfortable if the researcher collecting biopsies was of the same gender, which the team and nursing staff were able to accommodate. The protocol initially included a greater number of smaller biopsies to minimize the size of potential scars. However, patients expressed that they would prefer fewer larger biopsies, and given the extent of scarring from their HS, they cared less about cosmetics than about reducing the number of incisions. Participants asked for choice in location of biopsies, the use of numbing medication, explicit collection of co-morbid conditions and lifestyle data, and the return of results. Participants improved the clarity informed consent form language and suggested using visuals to demonstrate the size of planned biopsies. The investigator revised the protocol to incorporate participants’ feedback, including proactive draping, fewer biopsies, participant input into biopsy site selection, elective numbing medication, choice of provider gender for adolescents, lifestyle surveys, and specified plan for the return of results.

II. The goal of the **2021 FSTH, *“Shining Light on HS: Research Results and Moving Forward,*”** was to report on overall progress in HS research to the community (scientists, providers and patients) and to return the results from two studies forecast at the 2018 FSTH – investigation of HS pathophysiology and of a Phase I treatment trial with brodalumab. Through these studies, RU investigators have developed new insights into HS pathogenesis that have led to greater mechanistic understanding of how HS evolves, with implications for treatment. RU investigators reported the new findings implicating cytokines and immune pathways and showed graphic clinical slides and pathology demonstrating the dramatic positive effects in the treatment study. Participants learned about the increased interest within the pharmaceutical industry in treatments for HS, including two new drugs in phase III trials and multiple other drugs under investigation. Attendees expressed hope that more effective HS treatments would be available soon. Patient testimonies highlighted the ongoing challenges in living with HS, and patient advocates shared resources.

Two hundred and thirty-four people of varied occupational backgrounds registered for the 2021 FSTH (Fig. 2). One hundred and twenty-three participants attended. Participants viewed the webcast from 34 states and 34 countries outside the USA. While all attendees from the prior 2018 FSTH were invited, patient turnout was lower than for the previous event.

Feedback from participants from the 2018 and 2021 FSTH included the theme of periodically returning to the town hall format for interim updates. One participant in the 2018 town hall noted, *“More events like these would be fantastic… We need to build stronger relationships between patients and doctors to help patients understand the level of research being conducted as many patients I talk to are frustrated and have little hope for an effective treatment.”* At both the 2018 and 2021 town halls in the webcast chat thread, participants (including clinicians) wanted more information about specific HS treatments, including surgical methods. This theme informed the design of the third town hall.

III. The town hall in **2023, “The Cutting-edge Management of Hidradenitis Suppurativa (HS) in Primary Care*,”*** showcased updates in surgical management of HS geared specifically toward clinicians. The event covered current technical best practices in the diagnosis, management, and treatment of HS, including demonstration of surgical techniques [24]. In particular, an in-office surgical procedure called de-roofing was demonstrated and discussed as a strategy for managing dermal tunnels, a typical HS skin lesion. Given the technical and graphic information shared at this event, outreach specifically targeted provider networks. Eighty-three people registered for the third town hall, the majority of whom were clinicians (Fig. 2). Thirty-eight attended. Many registered as physicians in specialties suitable to conduct the less complex HS-related surgical procedures. Twenty-seven percent of attendees returned evaluations, and 100% rated the event as excellent or very good. The program included patient advocates who highlighted the impact of the experience and treatment of HS and the need for effective, respectful care.

Registration and attendance data show that pharmaceutical company representatives also attended and participated in Q&A sessions. In all, 429 people registered for all FSTH (including 102 patients and patient advocates, 203 clinicians, 72 public health professionals, 60 members of the pharmaceutical industry, 36 scientists/researchers, and 58 others) of whom 348 attended (Fig. 2). Participants viewed the webcast from 31 states and four countries outside the USA.

### Spanning the translational spectrum

The incorporation of expertise, data, qualitative experience, and a broad range of interested/affected populations is intentional in the design of the town halls. In Table 2, the activities of the town hall meetings are aligned with the phase of the translational spectrum.

**Table 2.** Alignment of meeting activities at hidradenitis suppurative full spectrum town halls with the phases of the translational research spectrum [25] and links to publicly archived recordings of the meetings

	Full spectrum town hall (FSTH) activities aligned to the phases of the translational spectrum [25]		
FSTH session, date	T1/T2 preclinical	T3 clinical	T4 Health impact, public health, and policy
Shedding light on hidradenitis suppurativa (HS) May 21, 2018	Reviewed immunology, pathology, and current gaps in understanding the biological mechanisms underlying HS. Announced a new initiative by the RU Laboratory of Investigative Dermatology to investigate the biological mechanisms driving HS and define its immune pathways in a clinical disease pathogenesis research protocol	Clinical overview: review of HS symptoms, presentation, histology; limitations of current treatment (one drug FDA-approved for HS, only somewhat effective). Announced a planned therapeutic trial for HS at RU based on emerging insights into HS biology (Phase I drug protocol for a drug currently FDA-approved for plaque psoriasis, brodalumab). Attendees invited to join a Community Engagement Studio to refine protocol design and study materials	HS Patient advocate described engagement with the community: advocacy organizations, primary care practices, mental health resources, and community networks, education and support programs. She also described her personal journey with HS. Patient panel gave testimonials about their experience living with and seeking care for HS, impact on quality of life, and emotional health. Limited availability of treatment options, high cost of the sole approved biologic; off-label anecdotes about other immunomodulators not FDA-approved for HS and not covered by insurers; treatment access issues
Community Engagement Studio [23] (FSTH follow-up activity) September 5, 2018		Engagement of 10 patients (8) and family members (2); feedback used to tailor the design of protocol procedures, informed consent, and clinical operations	
Shining light on hidradenitis suppurativa (HS): research results and moving forward June 24, 2021	Return of scientific results: New disease models, roles of cytokines, discovered as a result of the studies announced at the first FSTH [26,27]	Clinical updates on RU Phase I clinical trial of brodalumab; return of aggregate results, showing promise [26,27]. Discussion of two new drugs in phase III trials, and multiple others under investigation	Return of results to the broader HS community through the HS Foundation, FSTH recording posted online; through RU and parallel studies elsewhere, acceleration of research interest and disease understanding of HS; multiple potential treatments under development. HS patient advocate and patient perspectives express hope
Cutting-edge management of hidradenitis suppurativa (HS) in primary care June 8, 2023	Scientific updates about disease biology, immunology, mechanisms driving epithelial tunnels, physiology	Updates on drug trials: Phase 3 clinical trials completed for two new drugs targeting IL-17, secukinumab* and bimekizumab**, both awaiting FDA approval. Review of the persisting challenge of subcutaneous tunnels in HS and effectiveness of surgical approaches	Dissemination: video demonstration of *best practices* for surgical and therapeutic management of HS symptoms Clinician/surgical panel Q&A on implementing technique Reinforce awareness for providers and how to diagnose; use tools for assessment Quality of life screening Symptom/pain assessment and management Patient advocate perspective highlighted frustrations with misdiagnosis and delays and importance of provider education and awareness
Future meeting template	Plain language updates to mechanistic understanding of biology and immunology	Updates in clinical management, clinical trials, and drug approvals for new treatments	FDA advisory panel outcomes impact policy, insurance coverage Patient panel, perspectives

* Secukinumab received FDA approval for use in moderate to severe HS on 10/31/2023. ** Bimekizumab received FDA approval for use in moderate to severe HS on 11/20/2024. RU = Rockefeller University; FDA = Food and Drug Administration; FSTH = full spectrum town hall; Q&A = question and answer.

## Discussion

Three FSTHs provided opportunities for multi-stakeholder engagement about HS and made progress toward the intended impacts as described in the causal pathway (Fig. 1):

### Greater empathy for patients’ lived experiences

Patient testimonies were integral to all three events and provided an opportunity for researchers and clinicians to learn about patient priorities and lived experiences. Provided with a space to voice their experience and hear from others with HS, patients described feeling validated and connected to a wider community with similar experiences (Table 1). Patients viewed the testimonies as countering shaming or blaming remarks from doctors who lacked knowledge and empathy for HS, and to correct their misconceptions (see Table 1).

One of the FSTH speakers, a clinician-researcher already involved with HS advocacy, was further moved by the patient testimonies and FSTH format and extended the impact to a national conference:

*The first Town Hall changed the way I think about communicating HS research to the world. The AAD (*American Academy of Dermatology) *now offers support for invited patients to come and speak to dermatologists at their annual meeting, and the feedback has been fantastic. I think I wouldn’t have been such a proponent of that if we hadn’t had the Town Hall and I saw the impact of the people on the stage conveying different elements of the same message*. – Dr Michelle Lowes, dermatologist, HS Foundation Director and co-organizer of the HS FSTH.

### Patient-centered research

The FSTH directly affected the patient-centeredness of the HS protocols conducted at RU, through the subsequent CES and changes to the protocol according to patient feedback. Patient voices served as a general call for more patient-focused research on HS by humanizing the people impacted by the research and treatment gaps for HS (Table 1). By providing a forum for scientific investigators to present the latest research directly to the HS community in plain language, and hear from practicing clinicians and patients, FSTH invite greater clinician and patient collaboration in the scientific investigation of HS.

### Increased patient trust in research

Long-term trust is difficult to measure; however, patient participants expressed appreciation for HS researchers and a great willingness to engage: at the 2018 event, 143 attendees entered 745 comments in the webcast chat over the course of 90 minutes. Attendees volunteered for the CES and subsequently in research studies, returning to the 2021 and 2023 FSTHs to hear research results and updates.

The participation of patients was lower in the second and third town halls than anticipated. The organizers invited previous attendees and HS patient networks but engaged less actively with HS advocacy groups on social media. The third town hall was promoted largely to clinicians. The lower participation of patients over successive town halls speaks to the need for continuous reinforcement of intentional outreach to all stakeholders, especially patients, to sustain their engagement in translational research.

### Acceleration of discovery to impact

The FSTH coincided with the rise in availability of biologic therapies and a groundswell of interest and accelerated discovery in HS at RU and beyond. Following the initial FSTH, the RU investigators (JF, JK) initiated a series of HS protocols and produced numerous scholarly publications about HS. The studies conducted after the 2018 meeting provided new insights into disease biology and pathogenesis, response to biologic therapies, and contributed to the accelerating body of evidence driving new effective treatments for HS. A coarse measure of research into HS overall is the number of HS-related studies registered in clinicaltrials.gov in the 6 years preceding the 2018 FSTH and the 6 years from 2018 to the present (40 vs 126) and the number of publications concerning HS added to PubMed in the same time periods (742 vs 2772). The FSTH played a local role in accelerating translation at a moment of global interest in translation for HS.

### Improved diagnosis and treatment

HS patients repeatedly spoke about their negative interactions with medical providers. Participant evaluations from the first two FSTH specifically asked for information about surgical treatments, and providers expressed interest in learning surgical techniques to manage HS symptoms. The third FSTH was designed to address this gap in clinical understanding and care and specifically targeted medical providers who made up the majority of attendees. In the evaluation of the third FSTH, one participant said, “Everything presented was helpful and added valuable information to my knowledge as a nurse.” Since the first FSTH, two new drugs have been approved by the Food and Drug Administration for the treatment of HS: secukinumab on 10/31/2023 and bimekizumab on 11/20/2024. While the FSTH were not directly related to the approvals of these drugs, the work of RU investigators (JF, JK) contributed insights into underlying HS biology that continues to advance understanding and treatment.

### Limitations

Evaluation of the FSTH had limitations. The town hall online registration process collected information about attendees’ provider or non-provider roles without collecting medical (HS status) or demographic characteristics that would have enriched the analysis. As HS disproportionately affects underserved groups, and nonWhite patients are marginalized in HS care and research, it is especially important that we track these data consistently in all future FSTH. The project did not prospectively define how to measure trust/distrust, shame, empathy, clinicians’ fund of knowledge, or other concepts; organizers sought to minimize the burdens to attendance when first “meeting” this population. Nonetheless, that information would have been useful in measuring impact. The response rate for post-event evaluations was low (≥34%), likely due to delays in deployment. Eliciting feedback from attendees by providing a survey link in real-time during the seminar and providing a modest incentive for completion could enhance response rates going forward. Adding pre- and post-presentation surveys of knowledge for clinician-attendees would help to track comprehension and learning, and to estimate the impact of FSTH on diagnosis and treatment in practice.

Despite these limitations, the FSTH created a forum for patients, clinicians, scientists, and other interested individuals to engage and build a shared understanding of the process of full spectrum translational research. Each event incorporated patient perspectives and featured the patient voice. The FSTH laid the groundwork for the subsequent CES for an HS protocol and directly influenced the design of trials. Participants received meaningful updates about the impact of patient participation on progress in understanding biological mechanisms of disease and advancing treatment. Additionally, clinicians received information they can implement in their practices to improve the diagnosis and treatment of HS.

### Conclusion

These HS FSTH provide a useful model for increasing academic/community stakeholder engagement concerning a disease for which there are significant gaps in knowledge at several phases of the translational spectrum, and for which delays in diagnosis and treatment have left patients less trusting of research and healthcare at large. The partnering institutions leveraged their respective networks of partners to bring together the full range of stakeholders as both speakers and audience members. Patient voices and scientific and clinical advances shared the stage, and feedback from the range of partners suggests progress toward meaningful goals including enhancing patient-centered care for people living with HS. The content of the successive events builds on the content and feedback from the prior events, and each event offered concrete, actionable information, resources, and strategies to address stakeholder needs beyond the duration of the event.

## Supporting information

Hashemi-Arend et al. supplementary material Hashemi-Arend et al. supplementary material

## Supplementary material

The supplementary material for this article can be found at https://doi.org/10.1017/cts.2025.62.
