# Supplementary material for: Full spectrum town halls for hidradenitis suppurativa: A model for advancing clinician–patient–researcher engagement in clinical and translational research
Source: J Clin Transl Sci. 2025 Apr 11;9(1):e96. doi: 10.1017/cts.2025.62 (PMC12089856; doi:10.1017/cts.2025.62)
Supplement: Hashemi-Arend et al. supplementary material [file S2059866125000627sup001.pdf]

Supplementary Table S1. Additional Participant Comments

Comments	Theme
I hate that they say smoking & overweight I did not smoke at 11 years old, never been overweight	Frustration with doctors
I know. I say the same thing. I know people who never smoked and they get lesions.	
I live in [state] and am very isolated - also a veteran with PTSD, Are others here in HS groups on FB?	Psychosocial impact of HS
I would like to know more about the mental effects. I've had depression and anxiety since I was little.	
No one in my life has this or ever heard of it. It can feel very isolating and it's easy to scare yourself	
Has anybody gotten to the point of having surgery and/or laser? If so, did it work well for you?	Treatment and Symptom Management
I had surgery on my arm. Long recovery time (about 8 months) because my skin graft failed, but no problems since. No pain or issues since.	
Had laser surgery and it still came back like 10yrs later	
Antiandrogens caused a lot of weight gain for me... best thing so far has been the long-term antibiotics...Spearmint tea has also helped me, but the pain is real and the drainage...I hope that a cure can be found for this one day...#staystrong	
AIP - autoimmune protocol - it has changed my life!	
Antibiotics have caused severe stomach pains so I’ve gone off.	
Humira did not work for me messed up my blood and made HS worse	
Minocycline is the only antibiotic I actually had good results with.	
I'm having good results with Doxycycline (been on it for about 4 months now) legions & tracks have reduced a lot; hardly any drainage anymore; I'm worried what will happen when I have to cut it back or stop altogether.	
It's comforting to "be around" people who also struggle with this disease.	Impact of Town Hall
Had no idea so many people suffer from this. I’m so glad we’re all here.	
What a very productive, very open, very empathetic HS panel discussion! HUGE THANK YOU to all presenters, organizers, supporters, attendees in person and online! From me with love always to all touched by this terrible disease	
Thank you, everyone, conference personnel and attendees. I will sleep better tonight!	

I would love to be a part of a research team...this dreadful disease needs research! I was embarrassed for so long until I went to a doctor...imagine how many people are out there still suffering in silence	Interest in Research
Wish I lived closer to be able to participate in a study 😢	
It's true, research into HS is exploding. And we are different than a lot of previous conditions in that the patient voice is poised to unite and be heard from the start of this increased interest.	
I am very hopeful!! We will have a cure soon. Research and awareness has quadrupled in the last 10 years.	

Acronyms : HS=Hidradenitis Suppurativa, FB=Facebook, PTSD=Post Traumatic Stress Disorder
